# EVALUATING THE FEASIBILITY AND ACCEPTABILITY OF THE WITS WELLNESS COGNITIVE HEALTH PROGRAM

**DOI:** 10.1093/geroni/igac059.1686

**Published:** 2022-12-20

**Authors:** Julie Bobitt, Laura Payne, Neha Gothe, Chelsey Byers, Molly Hofer

**Affiliations:** University of Illinois at Chicago, Chicago, Illinois, United States; University of Illinois Urbana-Champaign, Champaign, Illinois, United States; University of Illinois at Urbana Champaign, Urbana, Illinois, United States; University of Illinois Extension, Champaign, Illinois, United States; University of Illinois Extension, Chicago, Illinois, United States

## Abstract

About 11% of U.S. older adults are at risk for or have subjective cognitive decline. Although some factors that affect brain health cannot be changed, research indicates lifestyle changes (i.e., physical activity, social engagement, heart-healthy diet) can delay or reduce cognitive decline. Drawing from existing research, UI Extension developed Wits Wellness, a 12-week program designed to enhance brain health among people ages 50 and older. Wits Wellness addresses multiple factors that affect cognitive health (e.g., physical activity, stress, sleep, social isolation, diet). Using a two-arm randomized control trial, 285 participants (mean age = 66.6 years, 24 males) were randomly assigned to the Wits Wellness intervention (in person or virtual) or a waitlist control group. Summative program evaluation surveys indicated adherence to the intervention was high, with 68% of participants attending 9 or more of the 12 sessions. Program acceptability was high, with 82.7% evaluating the program as great to excellent, and 90.8% reporting great to excellent interaction with program leaders. Approximately 63.6% of participant write-in comments included positive statements about the program challenging their brains, and 59.1% indicated the program positively affected participants’ socialization. For program feasibility, 90.4% rated the 60-minute weekly sessions ‘just right’ for program length, while 66.3% rated the 12-week program duration ‘just right’ and 27.7% rated it ‘too long’. Thus, preliminary results suggest that Wits Wellness delivered both in-person and virtually was well attended and highly acceptable in the community setting. Future studies will examine the program’s efficacy in improving physical and mental health related outcomes.

